# Supplementary material for: Dysregulated S100A9 Expression Impairs Matrix Deposition in Chronic Wounds
Source: Int J Mol Sci. 2024 Sep 16;25(18):9980. doi: 10.3390/ijms25189980 (PMC11432490; doi:10.3390/ijms25189980)
Supplement: Supplementary file 1 [file ijms-25-09980-s001.zip › ijms-3193288-supplementary.pdf]

Supplementary Materials

Supplementary methods:

Wound healing studies in mice

Full-thickness wounds (6mm in diameter) were generated under anesthesia on both sides of the back of C57BL/6 mice, db/db mice (13 weeks old) and mice with iron overload. In selected groups of db/db mice treatment with paquinimod (a gift from Active Biotech, AB, Lund, Sweden) at a concentration of 20 mg/kg/d was started two days after wounding. At indicated time points post wounding wounds were photographed and harvested for further analysis. To determine wound closure photographed wound areas were quantified using ImageJ software.

Macrophage generation

Macrophages were generated from bone marrow cells that were flushed out of trimmed leg bones from WT mice using RPMI 1640 medium (ThermoScientific, Karlsruhe, Germany) and a 25-G needle. Red blood cells were lysed before culture. Bone-marrow cells were differentiated to macrophages at 1×10^6 cells/ml in RPMI plus 10% FCS, 1% penicillin/streptomycin and 5 ng/ml M-CSF (Biolegend, San Diego, US) for 3 days.

Ex-vivo skin culture

To generate ex-vivo skin culture, mice were sacrificed, and skin prepared carefully removing the dermal fat from the dermis. The skin was incubated in DMEM medium supplemented with 10% FCS. Mouse skin was then stimulated ex-vivo with 10 ng/ml TNFα or 10ng/ml IL-1β together with 10 ng/ml TNFα (TI), 1 µg/ml insulin, 25mM glucose, 50µM $FeSO_4$ or 100µM $FeSO_4$. The skin was cultured for 24 hours at 37°C, 5% CO_2. To receive epidermis for PCR analysis skin was transferred in trypsin (0.25%) and incubated overnight at 4°C. At the next day the epidermis was separated from the dermis and used for RNA isolation.

Tissue staining

ECM deposition was detected by Masson trichrome staining. Mouse S100A9 expression was detected by an anti-S100A9 antibody (R&D, Wiesbaden, Germany) and an Alexa-Flour-546-conjugated anti-goat-antibody (Thermo Fisher Scientific). Human S100A9 was stained by anti-

S100A9 (R&D) and anti-sheep-biotin ((Thermo Fisher Scientific) followed by streptavidin-Alexa647 (Thermo Fisher Scientific). Images were captured using KEYENCE BZ-9000 fluorescence microscope (Keyence GmbH, Neu-Isenburg, Germany).

RNA preparation and quantitative real-time PCR

Total RNA from homogenized wounds, epidermis and cells was isolated using the Relia Prep RNA Tissue Miniprep System (Promega, Walldorf, Germany) according to the manufacturer′s protocol. cDNA was generated from a total of 500 ng RNA with LunaScript RT Supermix (NEB, Frankfurt a.M., Germany) as described in the manufacturer′s protocol. Quantitative real-time PCR was performed with LunaUniversal qPCR Mastermix (NEB) according to the manufacturer′s instructions (see Table S1 for primer sequences). All PCR products are intron-spanning. Quantitative gene expression was calculated from standard curve of cloned cDNA and normalized to the reference gene RS36.

Genome-wide expression analysis

RNA integrity and concentration were examined on an Agilent Fragment Analyzer (Agilent Technologies, Palo Alto, CA, USA) using the HS RNA Kit (Agilent) according to the manufacturer’s instructions. Microarray analysis was conducted at the Core Unit DNA Technologies (Faculty of Medicine; Leipzig University). cRNA was prepared from 100 ng of total RNA hybridized to Gene Chip Clariom S arrays (Thermo Fisher Scientific, Karlsruhe, Germany) according to the manufacturer’s instructions. The arrays were scanned with a third generation AffymetrixGeneChipScanner 3000. For data analysis, Affymetrix Gene Chip data were extracted from fluorescence intensities and were scaled in order to normalize data for inter-array comparison using Transcriptome Analysis Console (TAC) 4.0.2 software according to manufacturer’s instruction (Thermo Fisher Scientific).

Supplementary figures:

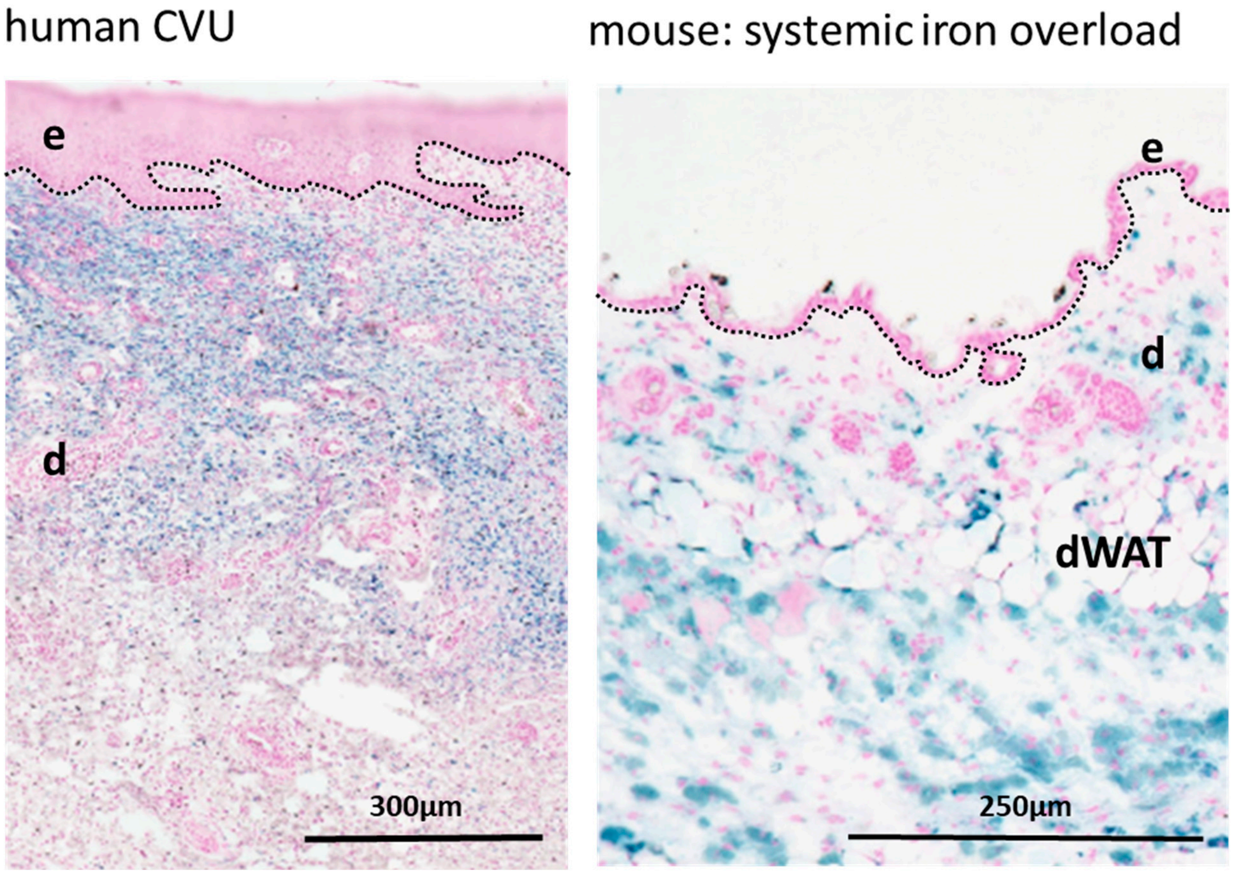

Supplementary Figure S1. Iron deposition in human chronic venous ulcer (CVU) and in skin of mice with systemic iron overload.

Cryo sections of skin wound margin from patients with CVU and cryo section of skin of mice with systemic iron overload were stained with Prussian blue to visualize iron deposition (in blue) in tissue. Level of iron deposition and distribution of iron within the tissue are similar in human CVU and mouse skin sections. e = epidermis, d = dermis, dWAT = dermal white adipose tissue, dotted line marks border between epidermis and dermis.

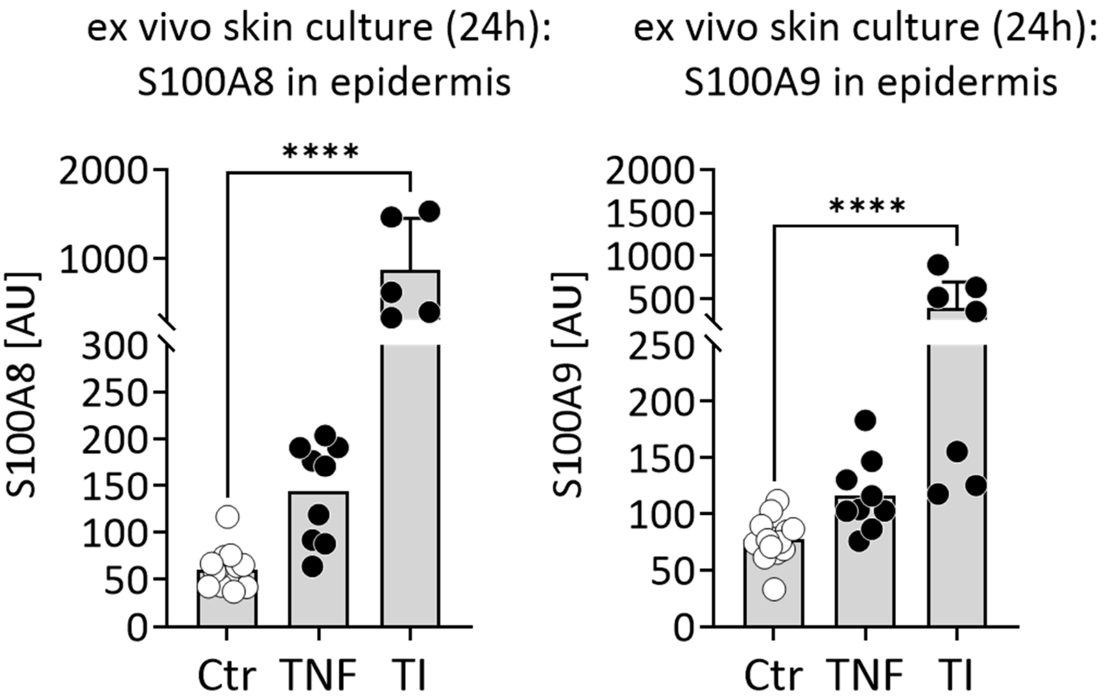

Supplementary Figure S2. Epidermal expression of S100A8 and S100A9 in skin cultures after stimulation with TNF and TNF/IL-1ß.

Skin of WT mice were cultured ex vivo and stimulated for 24 h with either 10 ng/ml TNF or with 10 ng/ml TNF together with 10 ng/ml IL-1b (TI) or left unstimulated (Ctr). After 24 h skin was harvested and epidermis separated from dermis to determine relative gene expression of S100A8 and S100A9 in the epidermis by quantitative PCR. Each dot represents one independent skin culture with skin samples isolated from 4 mice. Ordinary one-way ANOVA with multiple comparison to ctr. *P< 0.05, **P< 0.01

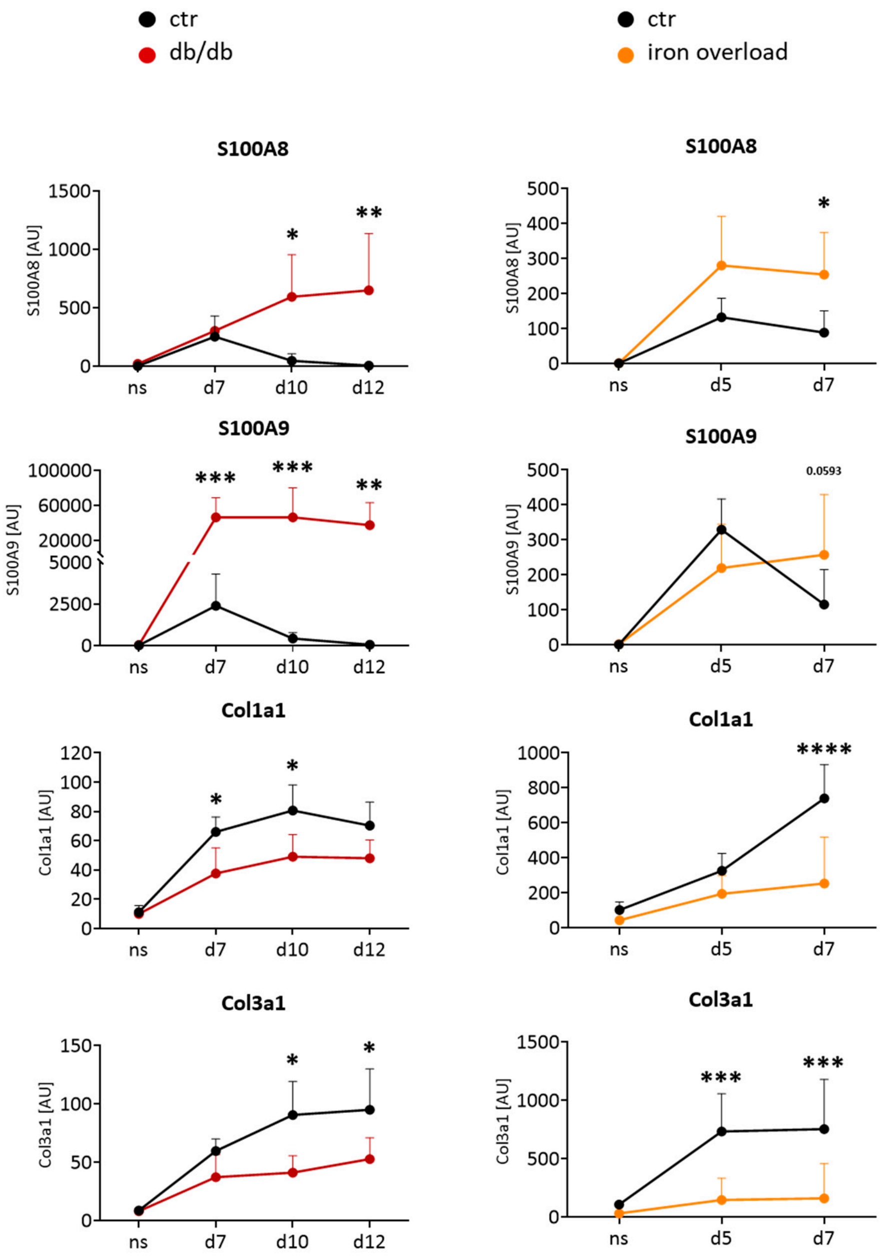

Supplementary Figure S3. Expression of S100A8 and A9 and of Col1a1 and Col3a1 in wounds during the course of wound healing in db/db mice and mice with iron-overload.

Db/db mice and iron-overloaded mice and their corresponding control mice were wounded with 6 mm punch biopsies. Relative gene expression of S100A8, S100A9, Col1a1 and Col3a1 was detected by quantitative PCR in wounds of the mice at the time points indicated. In control mice expression of collagens increases when expression of S100A8/A9 declines. In contrast, in db/db mice and in iron-overloaded mice high expression of S100A8/A9 persists in the wounds while at the same time induction of collagen expression is attenuated. Graphs contain data + statistics presented in the manuscript in Fig.1D/H, Fig.2D, and Fig.3A.

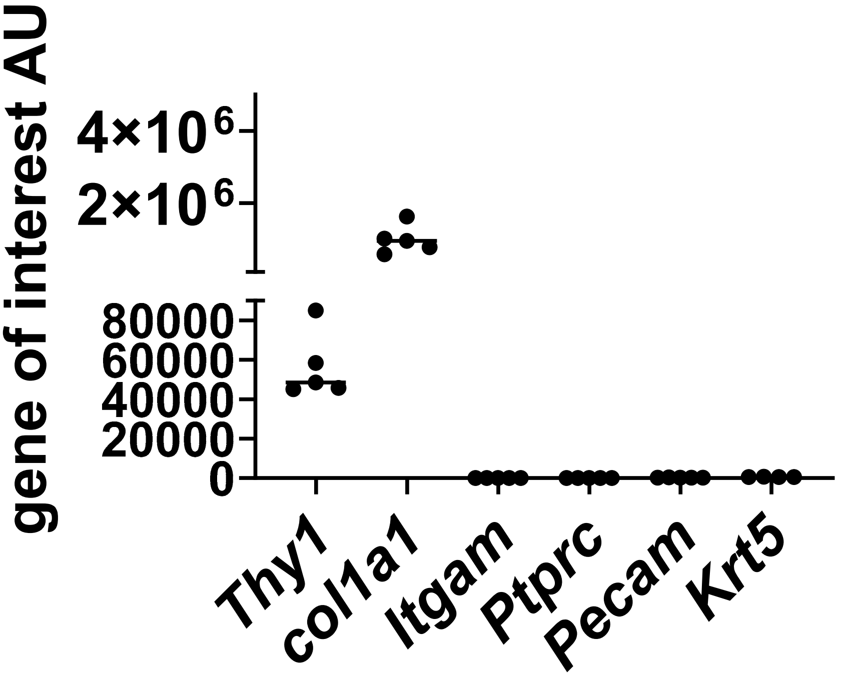

Supplementary Figure S4. Purity of isolated wound fibroblast from db/db mice.

Wounds were digested and skin fibroblasts were isolated by negative selection. Myeloid cells were removed by magnetic cell separation using CD11b+ Cell Isolation Kit. PCR analysis confirmed the purity of the cells. Endotheial cell marker (*Pecam*), keratinocyte marker (*Krt5*), marker for inflammatory cells (*Itgam, Ptprc*), fibroblast marker *Thy1* and *col1a1*. Each point represents cells from one mouse.

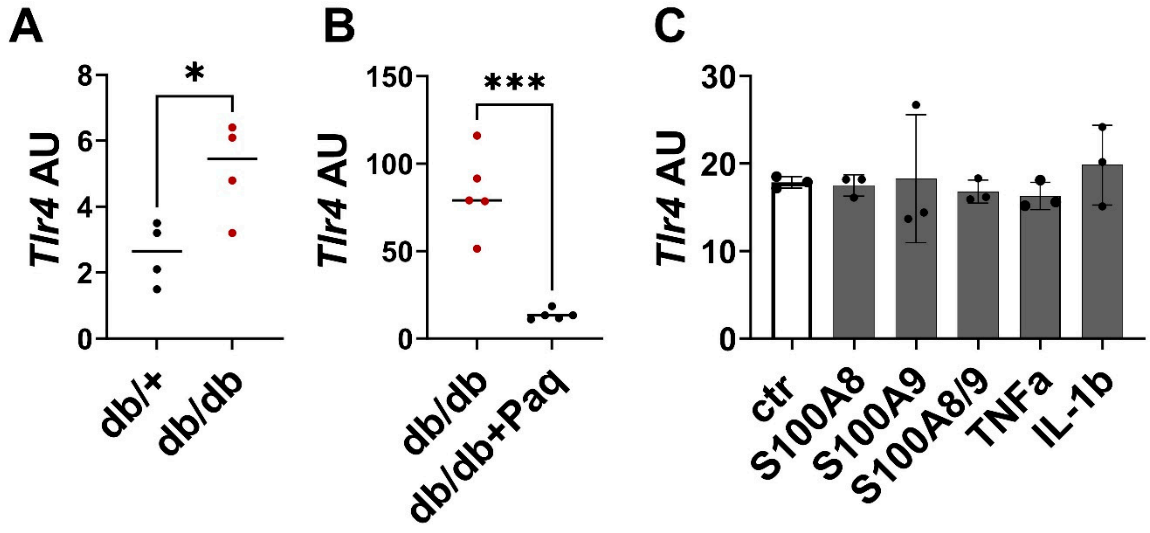

Supplementary Figure S5. Tlr4 expression is increased in diabetic conditions.

A) Fibroblasts were isolated from skin of db/db and control mice. B) Db/db mice were wounded with 6mm punch biopsies. Paquinimod (Paq) was applied 2 d after wounding (db/db+Paq). Fibroblasts were isolated 10 days after wounding. **C)** Dermal fibroblasts were stimulated with indicated mediators for 24h. **A-C)** Relative expression of Tlr4 detected by quantitative RT-PCR. Each dot represents one mouse or cells from one mouse. Mean (A/B) or Mean +/- standard deviation (C) is indicated. A/B) Unpaired t-test *P< 0.05, ***P<0.001, C) Ordinary one-way ANOVA with multiple comparison to ctr.

Supplementary Table S1: Primer sequences

Gene	Accession number	forward primer	reverse primer
Col1a1	NM_007742.3	TGTGCCACTCTGACTGGAAG	TTCTTTTCCTTGGGGTTCG
Col3a1	NM_009930.2	CCTTGGTCAGTCCTATGAG	CAGGAGCAGGTGTAGAAG
Il-1b	NM_008361.3	GACAACTGCACTACAGGCTCC	AGGCCACAGGTATTTTGTCG
Mmp3	NM_010809.1	CTATACGAGGGCACGAGGAG	TCTTCCTGGGAAATCCTGG
Mmp9	NM_013599.5	TAGATCATTCCAGCGTGCCG	GGACACATAGTGGGAGGTGC
Mmp10	NM_019471.3	TGAAGTCCAAGCAGGCTACC	TGGCATTGGGGTCAAACTCG
Mmp13	NM_008607	TTGATGCCATTACCAGTCTCC	TGGTTCTCAGAGAAGAAGAGGG
Plod2	NM_001142916.1	TCCTGATGGGTACTATGCTCGCTCT	CGGAGTAGGGGAGTCTTTTTCCCTT
Rs36	NM_007475	GGACCCGAGAAGACCTCCTT	GCACATCACTCAGAATTTCAATGG
S100A8	NM_013650	ACTGAGTGTCCTCAGTTTGTGC	CCCTAGGCCAGAAGCTCTG
S100A9	NM_009114	ACTCTAGGAAGGAAGGACACCC	TACACTCCTCAAAGCTCAGCTG
Tnfa	NM_013693.3	CTTAGACTTTGCGGAGTCCG	ACAGTCCAGGTCACTGTCCC
